# Are Guideline Recommendations on Supportive Nutrition and Exercise Therapy for Cancer Patients Implemented in Clinical Routine? A National Survey with Real-Life Data

**DOI:** 10.3390/nu15143172

**Published:** 2023-07-17

**Authors:** Luisa M. Hardt, Hans J. Herrmann, Dejan Reljic, Peter Jaensch, Jürgen Zerth, Markus F. Neurath, Yurdagül Zopf

**Affiliations:** 1 Hector-Center for Nutrition, Exercise and Sports, Department of Medicine 1, University Hospital Erlangen, Friedrich-Alexander University Erlangen-Nürnberg, 91054 Erlangen, Germany; hans.hermann@uk-erlangen.de (H.J.H.); dejan.reljic@uk-erlangen.de (D.R.); yurdaguel.zopf@uk-erlangen.de (Y.Z.); 2 Department of Medicine 1, University Hospital Erlangen, Friedrich-Alexander University Erlangen-Nürnberg, 91054 Erlangen, Germany; 3 IDC Research Institute, SRH University Wilhelm Löhe, 90763 Fürth, Germany; peter.jaensch@srh.de (P.J.); juergen.zerth@ku.de (J.Z.); 4 Faculty of Social Work, Catholic University Eichstätt-Ingolstadt, 85071 Eichstätt, Germany

**Keywords:** healthcare research, cancer patients, tumor cachexia, nutrition therapy, exercise therapy, supportive cancer therapy

## Abstract

Malnutrition and cancer cachexia are highly prevalent comorbidities of cancer, limiting patients’ quality of life and being relevant to prognosis. International and national clinical guidelines recommend supportive nutrition and exercise therapy for cancer patients. However, there is little current epidemiological evidence on the implementation of these guideline recommendations in clinical routine. To close this data gap, a national survey in Germany using an online questionnaire was conducted. There were 261 of a total of 5074 contacted hospitals and medical offices who participated in the survey (5.1% response rate). The data indicated that nutrition and exercise therapy for cancer patients is so far inadequately implemented, with 59% of the respondents reporting nutrition therapy as an integral part of oncological treatment, 66.7% having a nutrition specialist/team, and 65.1% routinely conducting a screening for nutritional status. Only half of the participants stated that there are defined goals in nutrition therapy. The majority of respondents (85.8%) generally recommend exercise therapy, but only a few of them provide specific offers at their own institution (19.6%) or at cooperation partners (31.7%). In order to implement the recommended combined nutrition and exercise therapy as part of regular care, there is a need for nationwide availability of multidisciplinary nutrition teams and targeted offers of individualized exercise therapy. Health policy support would be important to create the structural, financial, and staff conditions for appropriate guideline implementation in order to achieve the optimal treatment of cancer patients.

## 1. Introduction

In cancer patients, both disease- and therapy-associated influences often lead to cancer cachexia, which is associated with chronic inflammation and muscle breakdown [1]. Up to 75% of cancer patients suffer from unintentional weight loss and muscle wasting during the course of the disease, which can be substantial. Insufficient food intake, limited physical activity, and a catabolic metabolism caused by systemic inflammation interact synergistically. The progressive loss of muscle mass and muscle strength (sarcopenia) has a negative effect on the patient’s quality of life, morbidity, and mortality [2]. Twenty to twenty-five percent of cancer patients die as a result of cancer cachexia [3,4,5,6]. Hence, cancer cachexia and sarcopenia are relevant concomitants of cancer. Early individualized nutrition therapy together with adapted exercise programs can effectively stop fatal muscle loss or even promote muscle regaining, thus significantly improving cancer patients’ outcomes.

Therefore, they are important supportive therapy strategies that have found their way into international and national clinical guidelines for the care of oncological patients [3,7]. According to these guidelines, all patients should be regularly screened for malnutrition using validated screening tools, and nutritional therapy should be initiated if necessary. Moreover, the guidelines emphasize the relevance of combined exercise therapy for the effective maintenance or building of muscle mass and function [1,3,7].

Despite all this knowledge, published data show that the incidence of malnutrition and muscle loss in cancer patients remains very high [4,8]. Thus, the question arises whether the evidence-based recommendations of these international and national guidelines are being implemented in clinical routines at all. 

In order to close this data gap and to capture the real-life situation of the provision of supportive therapy to cancer patients, a national survey in Germany was conducted addressing practitioners in outpatient and inpatient settings and using an online questionnaire. The focus of the evaluation was to record the care structure of the participating institutions and to depict to what extent guideline recommendations are implemented in practice in a standardized manner. The survey data can contribute to identifying possible structural deficits in the supportive therapy care of cancer patients, as well as possible starting points for improving the nutrition and exercise therapy care situation.

## 2. Materials and Methods

In the first step, the nationwide relevant hospitals, hospital departments, outpatient clinics, and specialized medical offices were determined from the official registers. First, these relevant institutions and their contact persons were, respectively, informed of the survey and the evaluation process by a postal letter. In the next step, they were invited to participate by email and asked to complete the online questionnaire. Participants were then reminded of the survey three times also via email. The invitation email contained the link to participate in the online survey and an automatically generated transaction number (TAN) as a one-time password to avoid bias in the data and to ensure high data quality. This means that buffering and later continuation of the answering process was possible until the final dispatch of the answers. After that, the TAN expired and the given answers could no longer be edited. The survey was realized in the form of a specially developed online questionnaire, which contained 32 questions and was available for answering on the survey platform EvaSys (V.8.0 (2019), Electric Paper Evaluationssysteme GmbH, Lüneburg, Germany) for a period of 13 months. 

The questions were designed according to the recommendations of the nutrition guidelines [3,7]. The hypotheses for the questionnaire were derived from this in order to assess the actual care situation in everyday clinical practice. Standardized expert interviews were conducted to select the relevant topics and questions. The questions were carefully adapted to be clear and unambiguous, as well as selected and prioritized in such a way that answering the entire questionnaire would take no longer than 15 min. The questionnaire was evaluated in a pre-test before finalizing it for survey use. 

Closed single- and multiple-choice questions were supplemented in certain cases by open free-text answer options to enable respondents to complement and specify given answers. Answering all questions was voluntary, and the evaluation was carried out exclusively in anonymized and summarized form. After recording, checking, and cleaning, the data were subjected to univariate, descriptive, and hypothesis testing quantitative analysis procedures (X^2^-test, α = 0.05) using the statistical package SPSS (Version 26.0, Armonak, NY, USA: IBM Corp).

## 3. Results

### 3.1. Sample Description and General Data

**Figure 1:**
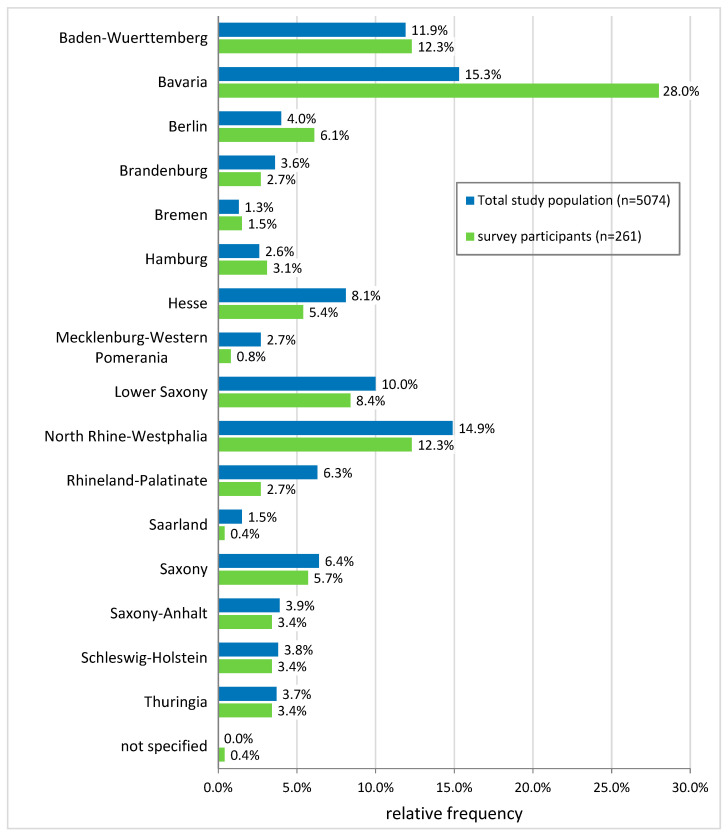
Distribution of the total study population and survey participants by federal states. Shown in blue is the proportion of participants by federal state in the initially contacted total study population (*n* = 5074). The green bars show the proportion of participants by federal state in the final study sample that answered the questionnaire (survey paticipants, *n* = 261).

For the online survey, the leading persons of a total of 5074 hospital departments and oncology-specialized medical offices/care centers were contacted, of which 261 answered the questionnaire (Figure 1). This corresponded to a response rate of 5.1%.

With Bavaria having a disproportionately high response rate of 9.4%, it had to be clarified whether, or to what extent, the increased participation of Bavarian facilities has an effect on a nationwide evaluation of the care situation in the sense of a “Bavarian bias”. Bivariate group comparisons of the Bavarian sub-sample against the other German participants with regard to the surveyed structural characteristics (i.e., service level, responsible body, department, and department size) showed no significantly different distributions within the sample (X^2^-test, α = 0.05), so that, with regard to the mentioned characteristics, the structural similarity of the participating hospitals/clinics and medical offices can be assumed and the hypothesis of a “Bavarian bias” can be rejected. 

With regard to the service level, university hospitals showed the highest response rate (12.4%), followed by outpatient medical offices/care centers (5.6%) and non-university hospitals (4.1%) (Table 1).

Regarding the hospital departments, surgery (22.3%), internal medicine (16.6%), oncology (15.8%), and gynecology (12.7%) together accounted for two-thirds of the participants. On average, the participants (*n* = 222) reported treating 248 oncology patients per quarter. Nearly 25% of the participants cared for patients exclusively as inpatients. 

### 3.2. Hypothesis Testing Analyses

In-depth analyses could not reveal any structural or contextual influencing factors. Thus, it can be assumed that there were no systematic differences with regard to region/federal state, service level, body of responsibility, size of the facility, or patient structure. 

### 3.3. Nutrition Specialist/Team

Two-thirds (66.7%) of the participants (*n* = 261) stated that they had a nutrition specialist or a nutrition team for the care of cancer patients. The responsible practitioners were mainly dieticians (75.3%), followed by physicians (44.8%), nutritionists/nutrition scientists (40.8%), nursing staff (35.6%), and medical doctors with additional qualifications in nutritional medicine (29.9%). Even though there was a trend towards more nutrition specialists/teams in larger clinical departments, there were no statistically significant differences regarding the department size. 

### 3.4. Screening for Nutritional Status

Screening for malnutrition in oncological patients was routinely carried out in 65.1% of the participants. Of these, one-third (33.3%) did not screen systematically and 34.1% only conducted the screening at the first presentation/admission. Seventy-seven-point-nine percent of the participants who regularly conducted a screening for malnutrition stated that clearly defined persons were responsible for the screening. Nurses/care staff were mainly (44.8%) responsible for carrying out the screening, and 30.6% nutritionists were involved and 23.1% physicians. Easy-to-perform standard procedures were predominantly used for the screening such as recording the body mass index (BMI) (80.5%) and validated screening instruments (67.2%) such as the Nutritional Risk Screening (NRS-2002), Malnutrition Universal Screening Tool (MUST), Mini Nutritional Assessment (MNA), or Subjective Global Assessment (SGA). Less frequently, body composition (e.g., using bioelectrical impedance analysis (BIA)) (19.09%), muscle strength as a functional parameter (e.g., hand grip strength) (7.5%), or anthropometry (e.g., upper arm/calf circumference) (4.6%) was measured. For most participants (68.9%), nutritional counselling was only provided if an obviously reduced nutritional status was identified. 

### 3.5. General Nutritional Recommendations and Dietary Counselling

**Figure 2:**
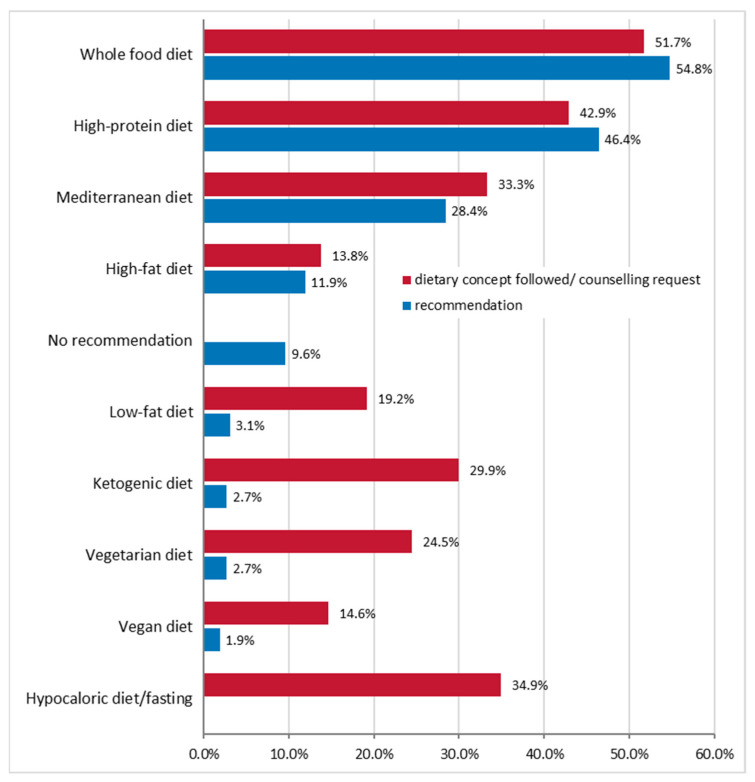
Recommendations given by the participants in the context of nutritional counselling (blue bars) vs. the patients’ request for counselling (red bars) on oral diet during cancer treatment (*n* = 261, multiple answers were possible).

In the context of dietary counselling, 54.8% of participants reported recommending a normal whole-food diet to cancer patients, 46.4% a high-protein diet, and 28.4% a Mediterranean diet. This seems to correspond to the patients’ wishes for advice. However, according to the survey participants, the cancer patients also had a significant preference for advice regarding a hypocaloric diet/fasting (34.9%), a ketogenic diet (29.9%), a low-fat diet (19.2%), a vegetarian diet (24.5%), and a vegan diet (14.6%) (Figure 2). 

As information options on the topic of nutrition for patients, the participants considered the distribution of printed information such as brochures (72%), as well as individual nutritional counselling (67%). Compared to hospitals (64.2%), individual nutritional counselling was offered significantly more often in outpatient medical offices/care centers (87.1%) (*p* = 0.011). 

### 3.6. Nutrition Therapy

About 59% of the respondents stated that nutrition therapy was an integral part of oncological treatment in their institution. The 41% denying participants had the opportunity to give corresponding reasons for this. The qualitative–explorative evaluation of the free answer option resulted in certain argumentation clusters: “due to the patient group/type of tumor/form of therapy”; “short length of stay in the facility”; “organizational reasons, especially resources, time, staff”; “no firmly implemented procedures/organizational structures”. Seven participants also indicated current efforts to establish or strengthen nutrition therapy structures in the future. 

In the realization of nutritional therapy, the entire spectrum from oral to enteral to parenteral nutrition seemed to be applied. About 75% of the respondents stated that they used patient-specific, individual combinations of these three forms of nutritional therapy. 

### 3.7. Nutrition Goals

**Figure 3:**
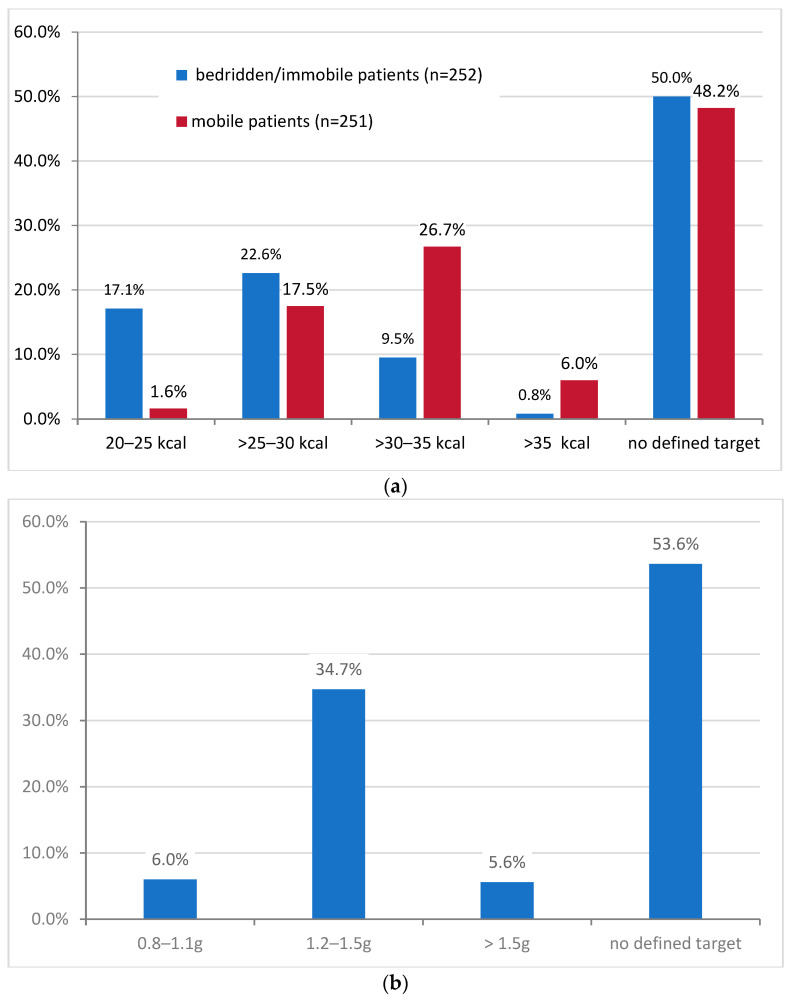
(**a**) Proportion of respondents who stated using a nutritional target for energy intake (in kilocalories (kcal) per kilogram of body weight per day) for bedridden (blue) and mobile (red) patients in their institution. (**b**) Proportion of respondents who reported using a nutritional target for protein/amino acid intake (in grams (g) per kilogram of body weight per day) for patients in their institution.

About half of the participants stated that they did not use any defined nutritional targets regarding energy (50.0% and 48.2% for bedridden and mobile patients, respectively) and protein/amino acid intake (53.6%) (Figure 3).

There was a high level of concordance regarding the (non-)existence of general nutritional targets for energy intake and a nutritional target for protein/amino acid intake. If defined energy targets existed, they usually did so for both the mobile and the bedridden patient groups. The existence of nutritional targets seemed to depend on the responsible department. They were most frequently stated in the departments of internal medicine, oncology, and radiotherapy. Furthermore, nutritional targets tended to be used more likely with increasing department size.

**Figure 4:**
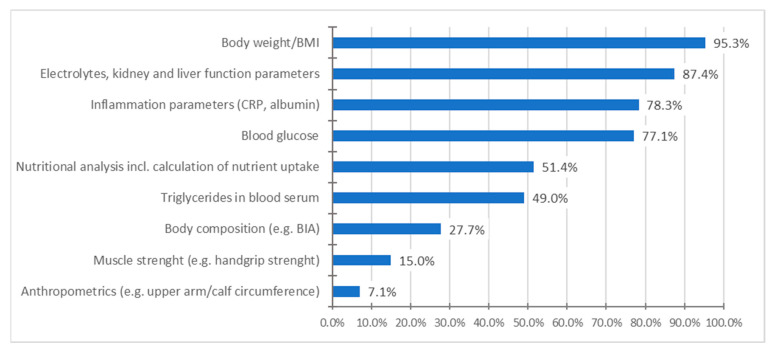
Diagnostic measures in the course of treatment.

The data on the diagnostic measures carried out in the course of the nutrition therapy (multiple answers were possible) showed a similar picture to the initial screening examination. Routine parameters (weight, BMI, laboratory parameters) were frequently assessed, whereas body composition (e.g., determination of muscle mass) and functional parameters (e.g., muscle strength measurements) were rarely assessed (Figure 4).

### 3.8. Physical Activity, Sports, and Exercise Therapy

Physical activity and functional capacity were predominantly (90.8%) assessed using the Karnofsky Performance Status scale or the Eastern Cooperative Oncology Group scale (ECOG). Sixty-point-five percent of the participants stated that they did not perform any specific performance tests. Among the tests that were used, spiroergometry with 18.4%, and the 6 min walk test with 18.0% were mentioned most frequently. The majority of respondents (85.8%) made a general recommendation about exercise and sports to their patients. A specific offer of targeted exercise therapy was provided by 19.6% of them at their own facility and by 31.7% at cooperation partners (e.g., sports clubs or centers). With regard to the exercise therapy offered, group courses dominated (81.7%). Regarding the training content, combined strength and endurance training was most frequently recommended (71.4%). More than half of the respondents (58.5%) did not give a specific recommendation.

## 4. Discussion

Efforts have long been made by nutritional medicine societies to raise awareness among treating physicians of the significance of the consequences of malnutrition and muscle wasting for the course of the disease and the quality of life of cancer patients. Meanwhile, convincing scientific evidence for the benefits of supportive nutrition and exercise therapy has been presented in national and international clinical guidelines [1,3,7].

Despite these efforts, the prevalence of malnutrition in cancer patients remains high [4]. The present survey showed that the guideline recommendations still need to be better implemented in the real-life setting in order to achieve evidence-based, optimal supportive therapy care for cancer patients.

According to the guidelines, validated screening for malnutrition and muscle wasting should be performed repeatedly in cancer patients at diagnosis and during the course of the disease [1,3,7,9]. In the present survey, one-third of the respondents stated that they did not perform screening at all and another third performed it only once at the first presentation. Only very few followed the recommendation to repeatedly evaluate the nutritional status during the course of the disease. Nursing staff (45%), followed by dieticians (31%) and physicians (23%) seemed to be responsible for the screening. It is possible that a clear assignment of the responsibility for screening could improve the detection of malnutrition. Our survey showed that nurses are the main screeners. In clinical routine, patient-relevant data such as diagnosis, weight, appetite, and unintentional weight loss are increasingly digitally recorded by nursing staff. At the same time, these parameters are also included in validated screening tools such as the NRS 2002. Thus, for an efficient screening, the patient data already digitally recorded could be used, possibly supported by artificial intelligence technologies (AI), as already done in other areas of medicine [10].

Body composition is more crucial for prognosis than total body weight [3], and reduced muscle mass is considered an independent diagnostic criterion of malnutrition according to the Global Leadership Initiative on Malnutrition (GLIM) criteria [11]. In nutritional assessment, it is, therefore, recommended to determine body composition (e.g., by means of BIA) [1,3,7,9]. However, this does not seem to be possible in a standard manner due to a lack of technical equipment. Accordingly, only 19% of the respondents stated that they can assess the body composition using a BIA device.

Early initiation of integrative nutrition therapy in parallel with anti-cancer therapy is essential [3,12,13], and combined nutrition and physical exercise interventions are most effective at the stage of pre-cachexia [14,15]. However, in the present survey, 68.9% of the participants stated that the consultation of the nutrition therapist only took place when there was an obvious nutritional disorder. It can, therefore, be assumed that the potential of early nutrition therapy is not optimally exploited at present.

To prevent malnutrition and muscle wasting, the guidelines recommend an adequate intake of energy and nutrients, especially protein/amino acids. For the daily energy intake, 25 kcal/kg body weight is recommended for bedridden patients and 30 kcal/kg body weight for mobile patients. To prevent muscle wasting, a daily protein/amino acid intake of 1.2–1.5 g/kg body weight (up to 2.0 g/kg body weight in severe cachexia) is recommended [3,7]. Our survey data showed that, if participants were using defined nutritional goals in their work area, these were predominantly in the guideline-compliant ranges. In the case of defined nutritional targets, most participants conducted appropriate nutrition therapy reporting to use 25–30 kcal/kg body weight as the nutritional target for the daily energy intake of bedridden patients (22.6%) and 30–35 kcal/kg body weight for mobile patients (26.7%), respectively. There were 34.7% using 1.2–1.5 g/kg body weight and 5.6% using >1.5 g/kg body weight as a nutritional target for daily protein/amino acid intake. However, about half of the participants did not use defined nutritional goals at all.

Only a targeted, individualized nutritional therapy adapted to the body weight and inflammatory status can improve the nutritional and muscle status and, ultimately, the prognosis of the patient. Uncontrolled intake of energy without a focus on the necessary proteins/amino acids increases the risk of progressive muscle loss and complications such as hyperglycemia, fatty liver, or refeeding syndrome [16,17]. As in other medical fields, standard operating procedures (SOPs) are shown to improve care and can enhance the quality of staff work. Therefore, it would be desirable that the recommendations of the nutritional guidelines are also incorporated into SOPs for the treatment of cancer patients [18].

In light of the stepwise scheme recommended in the guidelines [1,3,7,9], the survey result that 75% of the respondents used patient-specific combinations of oral, enteral, and parenteral forms of support when conducting nutrition therapy can be interpreted positively. Compared to inpatients, outpatients were more often offered individual nutritional counselling (64.2% vs. 87.1%; *p* = 0.011). However, malnourished inpatients should also receive individual counselling in order to receive nutritional therapy adapted to their disease process. In order to realize individualized nutrition therapy in all care settings, AI is increasingly being used here as well [19]. Whether the complex situation of cancer disease can be covered with this innovative possibility remains to be seen in the future.

Fortunately, the advice given of a normal oral diet during anticancer therapy is largely guideline-compliant with participants, who mainly recommended a healthy, nutrient-dense and protein-rich diet (whole-food diet, 54.8%; high-protein diet, 46.4%; Mediterranean diet, 28.4%; multiple answers were possible). Cancer patients often want to actively combat their cancer by changing their diet, and the present survey data showed that there was a relevant interest in restrictive diets (hypocaloric diet/fasting, 35%; ketogenic diet, 30%; vegan diet, 15%) on the patients’ side. Restrictive diets, however, carry the risk of malnutrition and muscle loss, so they cannot be generally recommended. Short-term fasting may have a positive effect on the side effects of chemotherapy, such as stomatitis, but not on tumor growth [20]. Fasting concepts should be adopted, if at all, only after individual counselling and under medical or nutritional therapy supervision [21]. For other elimination diets, such as the ketogenic diet, there is so far no clear evidence from high-quality clinical trials in cancer patients that it can reduce tumor growth or metastasis or improve the effectiveness of chemotherapy/radiotherapy [3,22,23]. As animal protein sources play an important role in supporting muscle anabolism under conditions of increased protein turnover and requirement, it is becoming increasingly evident that the restriction of animal proteins is inappropriate and even harmful during active cancer [24].

In summary, there is a huge need, as well as a considerable educational potential of dietary counselling for cancer patients. However, the existing dietary counselling offered in German hospitals and medical offices is currently not sufficient to provide adequate care for the patients.

Exercise and sports therapy, combined with nutritional therapy, provide enhanced anabolic stimuli to help maintain or build muscle mass and function and to counteract side effects such as fatigue [25,26]. Adapted exercise/sport programs are shown to be feasible in all stages of cancer and its treatment [25,26,27,28,29,30,31] and should be an accompanying component of any type of nutritional intervention [3]. The majority of respondents (86%) in our study generally recommended exercise and sports to their patients. However, specific therapy offers were provided by only 19.6% at their own facility and by 31.7% at cooperation partners (e.g., sports clubs and centers). Evidence shows that physical activity programs for cancer patients are actually taken up by less than half of the eligible patients [32]. The low participation could be due to disease- and therapy-related symptoms such as fatigue, pain, and physical weakness, which make it difficult for patients to complete the sport programs. Exercise modalities specifically tailored to the needs of cancer patients could increase acceptance among cancer patients [32]. For instance, the use of whole-body electromyostimulation (WB-EMS) [30,31] or very low-volume, high-intensity interval training (LOW-HIIT) has been shown to be feasible, safe, and effective, even in patients with advanced cancer [33]. However, the present study also showed that too few targeted training recommendations are still made by treating physicians. Early and extensive medical advice on the safety and benefits of these interventions can make an important contribution to ensure that patients take advantage of exercise therapy already during their anticancer therapy.

In order to be able to develop an individualized training/exercise program, the assessment of the patients’ performance status is crucial. However, more than half of the survey participants (60.5%) reported that they did not perform any performance tests. The majority (90.8%) used the Karnofsky- or ECOG-Performance Status scale to assess physical activity. These are established scores to broadly assess the physical condition and, thus, the health status of the patient and are classically used in oncology. To evaluate the compliance, feasibility, and effectiveness of exercise therapy interventions, it would be additionally necessary that performance tests, such as spiroergometry and the 6 min walk test, are conducted more frequently than it seems so far (18.4% and 18.0%, respectively) [1,7,9].

This nationwide survey in Germany representatively indicated that the implementation of the nutrition and exercise guidelines for oncology patients in the real-life setting still requires optimization—both in the identification of patients in need and in the implementation of a standardized evidence-based supportive therapy. Against this background, the question arises why evidence-based recommendations are not implemented in clinical practice.

A national, representative, cross-sectional survey from China (1732 participants from 51 clinics/hospitals) identified the lack of education and training of healthcare staff in the use of clinical guidelines as a key barrier to guideline implementation, while experience with evidence-based medicine in education and practice was significantly associated with self-reported guideline adherence [34]. Durán-Poveda et al. also confirmed in their survey that there is little knowledge among medical staff about nutritional guidelines and that, therefore, evidence-based nutrition therapy can be offered to cancer patients far too seldom [35]. Better education and further training of healthcare staff might, therefore, be an important target point to optimize guideline implementation in clinical practice.

The present study had limitations with regard to the response rate, as well as with regard to more detailed analyses. The chosen methodology of the online survey was intended to minimize barriers in the form of time and organizational effort for the participants. The response rate of 5.1% appears low, but is in line with comparable surveys such as the Italian study by Caccialanza et al. (2016), which also reported a response rate of 5.7% for their national, web-based survey among 2375 oncologists in a similar context [36].

In terms of time efficiency, the questions had to be carefully selected and priorities set. The focus was on a general status quo analysis of the nutrition and exercise therapy care of cancer patients in German clinics/hospitals and specialized medical offices/care centers, which enabled an actual target comparison with the guideline recommendations. More detailed and in-depth questions of future studies would be useful to gain a better insight into the corresponding processes and structures of nutrition and exercise therapy care for cancer patients to concretize improvement strategies for guideline implementation.

## 5. Conclusions

In the present study, we were able to show that structural and organizational hurdles need to be overcome in order to implement nutrition and exercise therapy guidelines for cancer patients in clinical routines. The education and further training of the medical staff seemed to be of central importance in this context. Moreover, the technical prerequisites (such as a BIA device) must be made available in order to be able to carry out the measurement of body composition/muscle mass required by the guidelines. SOPs on nutrition and exercise therapy integrated into cancer treatment could facilitate the work processes and ensure the application of evidence-based treatment. Greater health policy support should be given to hospitals and specialized oncology medical offices in order to implement these measures in a real-life setting.

## Figures and Tables

**Table 1:** Response rate by service level.

Service Level	Total Study Population	Survey Participants	Response Rate
Basic/regular/central service hospitals; maximum care hospitals	4000	164	4.1%
Universities	525	65	12.4%
Oncology-specialized medical offices/care centers	549	31	5.6%
No specification	-	1	-
Total	5074	261	5.1%

## Data Availability

The datasets generated and analyzed during the current study are not publicly available, but are available from the corresponding author upon reasonable request.
